# The effect of methadone on depression among addicts: a systematic review and meta-analysis

**DOI:** 10.1186/s12955-020-01599-3

**Published:** 2020-11-23

**Authors:** Masoud Mohammadi, Mohsen Kazeminia, Nasrin Abdoli, Behnam Khaledipaveh, Shamarina Shohaimi, Nader Salari, Melika Hosseinian-Far

**Affiliations:** 1 Department of Nursing, School of Nursing and Midwifery, Kermanshah University of Medical Sciences, Kermanshah, Iran; 2 Department of Psychiatry, Substance Abuse Prevention Research Center, Kermanshah University of Medical Sciences, Kermanshah, Iran; 3 Sleep Disorders Research Center, Kermanshah University of Medical Sciences, Kermanshah, Iran; 4 Department of Biology, Faculty of Science, University Putra Malaysia, Serdang, Selangor Malaysia; 5 Department of Biostatistics, School of Health, Kermanshah University of Medical Sciences, Kermanshah, Iran; 6 Department of Food Science and Technology, Ferdowsi University of Mashhad (FUM), Mashhad, Iran

**Keywords:** Methadone, Depression, Addiction, Systematic review, Meta-analysis

## Abstract

**Background:**

Opioids addiction and misuse are among the major problems in the world today. There have been several preliminary studies examining the effect of methadone on depression among addicts, however, these studies have reported inconsistent and even contradictory results. Therefore, the aim of the present study was to determine the effect of methadone on depression in addicts in Iran and around the world, using a meta-analysis approach.

**Methods:**

This study was a systematic review and meta-analysis including articles published in the SID, MagIran, IranMedex, IranDoc, Cochrane, Embase, ScienceDirect, Scopus, PubMed and Web of Science databases were searched systematically to find articles published from 2006 to March 2019. Heterogeneity index was determined using the Cochran's test (Qc) and I^2^. Considering heterogeneity of studies, the random effects model was used to estimate the standardized difference of mean score for depression. Subsequently, the level of depression reduction in Iran and worldwide in the intervention group before and after the testwas measured.

**Results:**

A total of 19 articles met the inclusion criteria, and were therefore selected for this systematic review and meta-analysis. The sample size of the intervention group in the selected studies was 1948. According to the meta-analysis results, the mean depression score in the intervention group was 26.4 ± 5.6 and 18.4 ± 2.6 before and after intervention respectively, indicating the reducing effect of methadone on depression, and this difference was statistically significant (*P* < 0.01).

**Conclusion:**

The results of the present study show that methadone significantly reduces depression in addicts. Therefore, regular methadone use can be part of a drug treatment plan.

## Background

Drug addiction and opioids misuse are among some of the key challenges and concerns of contemporary societies; they are even considered as the fourth major societal challenges after nuclear issues, population increase, and environmental pollution [1]. The addiction phenomenon initially reflects an image of a risky individual problem, while it is a social, economic, health, and security concern in different countries [2]. The prevalence of drug addiction is increasing rapidly in Iran and globally, with youth and adolescents making up a large percentage of drug addicts [3]. For instance, the rate of drug abuse has increased 3 times more than the population growth rate in Iran over the past 20 years [4]. Hence, it is important to control addition to opioid among the general population. Considering 1925 km of common border with Afghanistan and Pakistan, which are the major sources of poppy cultivation and opium and heroin production in the world, Iran is particularly vulnerable, since it is on the path of transit of opiates from these countries to Europe [1]. Heroin, for instance, is expensive and it is difficult to treat its addiction and break its defective cycle, and hence requires a comprehensive treatment system with different approaches of drug therapy, psychotherapy and rehabilitation. Prevention is logically the preferred option compared to treatments. Nowadays, the addiction problem is not only a family concern, but is also a sociodemographic challenge. It has negative effects on the mental health of the addict and addicts and their families [5]. One of the top priorities in today’s societies should be to identify addiction-related problems and plan to control and reduce the number of addicts [1].

Nearly 90% of opioid addicts have some form of mental disorder, among which are depression, antisocial personality disorder, and anxiety [6]. According to the diagnostic and statistical manual of mental disorders (4th edition)—text revision (DSM-IV-TR), depression is characterized by sadness, low self-confidence, and a lack of interest in any kind of daily activity and enjoyment. Depression can lead to a significant disorder negatively affecting one’s personal and social life, and employment. It also disturbs one's daily performance i.e. eating, sleeping and health [7]. Depression may be a sign of helplessness among addicts; it is considered as a barrier to effective behaviors to combat addiction or to exploit coping resources available to substance abusers [8]. The prevalence of major depressive disorder and minor depression among addicts are approximately 50–60% and 10% respectively [9]. Blanchard (2000) studied 872 methadone-treated patients and examined the presence of axis I and II disorders. He demonstrated that if an opioid addict had one of the first axis disorders, would need the drug addiction therapies, and psychotherapy, combined with methadone maintenance treatment (MMT) [10].

Methadone is used as one of the biological therapies for detoxification and maintenance treatment of heroin addiction and other opioid-like substances. The low cost of methadone, along with its high potency in controlling the physical and psychological conditions of opioid-like addiction, has made it a useful drug in the treatment of addicts [11]. Although MMT is considered a form of physical addiction to this drug, it is not considered equal to addiction, since by the regular use of the drug, the patient gets rid of euphoria, hangover and compulsive drug use. Such deification allows the patients to return to their communities and focus on other areas of life [12]. Previous research works have reported inconsistent results on the effect of MMT on addicts' mental health; for instance, some studies have shown that methadone-treated addicts have a high level of mental health problems compared to the general population and most have experienced mood and emotional disorders such as depression and anxiety [13]. In MMT, the substance is administered to patients in an oral syrup in a controlled manner. Experts believe methadone replacement reduces the prevalence of injection drug addiction and dangerous diseases such as AIDS, and on the other hand, breaks the link between drug addicts with drug dealers and decreases the likelihood of delinquency [1].

There have been several preliminary studies on the effect of methadone on depression in addicts, which yielded contradictory results. One of the applications of meta-analysis studies is to respond to these assumptions and resolve such contradictions. Therefore, the aim of the present study is to determine the effect of methadone on depression in addicts in the world using a meta-analysis approach.

## Method

This study was a systematic review and meta-analysis including articles published in databases of SID, MagIran, IranMedex and IranDoc and Cochrane, Embase, ScienceDirect, Scopus, PubMed and Web of Science (WoS) were searched in order to find relevant articles published from 2006 to 2019 in Persian and English language (In order to access quality articles and present more up-to-date results, the desired time frame was selected), matching the selected search keywords. These articles were obtained based on PRISMA statement and the initial article search process, removal of duplicate articles, review of articles in terms of relevance, as well as quality evaluation and finally final extraction of articles.

### Literature search

The search strategy involved a series of complementary search methods including a comprehensive search of key bibliographic databases and manual search of reference lists or citations follow-up of identified eligible articles and relevant reviews which would not be captured through the bibliographic databases search. Using relevant search terms developed from Medical Subject Headings (MeSH), Search terms to be used included the following: ‘Methadone’, ‘Depression’, ‘Opioid’, ‘Drugs’, ‘Addicts’, ‘Addiction’, ‘Drug Addict’, ‘Substance Use’ and ‘Drug Abuse’.

Words “AND” and “OR” were used in combinations to obtain more comprehensive articles, where “OR” was used for different common names of a disorder and “AND” was employed between words.

((((((((((Methadone’[Title/Abstract]) OR (Drugs’[Title/Abstract])) AND (Addicts’[Title/Abstract])) AND (Addiction’[Title/Abstract])) AND (Drug Addict’[Title/Abstract])) AND (Substance Use[Title/Abstract])) AND (Drug Abuse[Title/Abstract])) AND (Depression’[Title/Abstract])) OR (Psychopathy[Title/Abstract])) OR (Psychiatry[Title/Abstract])) OR (Psychology[Title/Abstract])))))))))).

### Selection criteria

Inclusion criteria included studies that examined the effect of methadone on depression among addicts, interventional studies, clinical trials, studies written in English language or with at least an abstract in English, similarly, the exclusion criteria included pre-prints, irrelevant articles (articles that have studied the effects of methadone on addicts but did not have a depression index), research works without sufficient data, duplicate articles, systematic reviews and meta-analysis works, cross sectional studies, case control studies, cohort studies, case reports, case series, letter to the editor.

### Quality evaluation

The Newcastle–Ottawa scale was used to assess quality, The Newcastle–Ottawa Scale (NOS) is a quality assessment tool is recommended by the Cochrane Collaboration [14]. The NOS assigns up to a maximum of nine points for the least risk of bias in three domains: (1) selection of study groups; (2) comparability of groups; and (3) ascertainment of exposure and outcomes for case–control and cohort studies, respectively [14], and 11 scores possible. Eventually, articles scoring < 5 points were classified to low quality. In this meta-analysis, all the articles that obtained five or more points were included**.**

## Data extraction

Data from all articles entered into the meta-analysis were extracted using a different pre-prepared checklist. The checklist included article’s title, first author's name, year of publication, place of study, sample size, sample size of intervention group, mean sample before and after intervention, and standard deviation of sample before and after intervention. The type of interventions mentioned in each study are reported in Table 1.

**Table 1:** Specifications of studies entered into the meta-analysis

Author, year, Reference	Place of study	Sample size intervention group	Mean ± SD of before	Mean ± SD of after	Method	Study design	Type of intervention	Quality
Lotfi [15]	Iran	10	28.7 ± 3.09	10.5 ± 5.58	BDI^1^	Clinical trial (with pretest –posttest and control group)	10 to 20 cc of methadone syrup per day for 12 weeks	High
Pournaghash [16]	Iran	50	6.91 ± 6.35	3.56 ± 3.7	GHQ-28^2^	Clinical trial	12 to 24 cc of methadone syrup per day for 12 weeks	High
Rezaei [17]	Iran	50	1.7 ± 1.3	0.35 ± 0.67	BDI	Randomized, controlled clinical trial	15 to 40 cc of methadone syrup per day for 6 weeks	High
Yaghoubi [18]	Iran	12	23.83 ± 2.24	17.33 ± 1.61	BDI	Clinical trial	Methadone syrup or tablets for 6 weeks	High
Ardani [19]	Iran	12	24.41 ± 10.5	11.16 ± 4.98	SCL-90-R^3^	Clinical trial	15 to 40 cc of methadone syrup per day for 9 weeks	High
Ahmadvand [20]	Iran	19	38.6 ± 10.59	28.22 ± 11.29	BDI	Clinical trial	Methadone therapy for 3 months	Medium
Hosseini [21]	Iran	107	26.03 ± 5.95	19.02 ± 7.23	BDI	Clinical trial	Methadone syrup for 6 weeks	High
Jondaghi [22]	Iran	10	17.26 ± 8.42	14.1 ± 3.95	SCL-90-R	Clinical trial	Methadone therapy for 12 weeks	High
Khaledian [23]	Iran	12	39.91 ± 4.33	32.08 ± 3.67	BDI	Clinical trial (pretest –posttest and control group)	Methadone syrup or tablets for 12 weeks	Medium
Jenaabadi [24]	Iran	19	32.38 ± 5.59	18.3 ± 3.03	BDI	Clinical trial	0.5 mg / kg methadone syrup for 12 weeks	Medium
Taimouri [25]	Iran	12	21.43 ± 3.14	16.54 ± 3.22	BDI	Clinical trial	Methadone syrup or tablets for 9 weeks	High
Saedy [26]	Iran	14	26.1 ± 16.4	16.6 ± 11.6	BDI	Clinical trial	0.5 mg / kg methadone syrup for 9 weeks	High
Yin [27]	China	1301	50.52 ± 10.99	48.22 ± 10.06	SDS^4^	Clinical trial	Methadone syrup or tablets for 1 months	High
Newville [28]	USA	100	41.6 ± 7.1	38.6 ± 7.6	BDI	Clinical trial	Methadone syrup or tablets for 3 months	High
Jovanović [29]	USA	69	27.46 ± 4.16	17.69 ± 3.68	BDI	Clinical trial	Methadone syrup or tablets for 6 months	Medium
Lin [30]	Taiwan	50	18.51 ± 2.61	2.49 ± 2.89	BDI	Clinical trial	0.5 mg / kg methadone syrup for 12 weeks	High
Silverman [31]	USA	24	28 ± 7	23 ± 3	BDI	Clinical trial	15 to 40 cc of methadone syrup per day for 12 weeks	High
Schreiber [32]	Israel	63	17.4 ± 6.2	11.8 ± 8.4	BDI	Clinical trial	10 to 20 cc of methadone syrup per day for 8 weeks	High
El Hage [33]	Canada	14	34.3 ± 1.5	17.3 ± 2.3	MADRS^5^	Clinical trial	15 to 40 cc of methadone syrup per day for 8 weeks	High

^1^Beck depression inventory

^2^Goldberg health questionnaire

^3^Symptom check list-90-revised

^4^Self-rating depression scale

^5^Montgomery-asberg depression rating scale

### Statistical analysis

Frequency, percentage and standard deviation indices were used to combine the reported results of all of the selected studies. To evaluate the heterogeneity of the research works, the I^2^ index test was used. Due to heterogeneity of the studies, the random effects model was used to amalgamate the reported results and conduct the meta-analysis. Low, moderate, and high heterogeneity were indicated by I^2^ < 25%, 25% < I^2^ < 75%, and I^2^ > 75%, respectively. *P* < 0.05 was considered as the significance level. The Egger’s test and corresponding Funnel plots were used to assess the publication bias.

## Results

**Fig. 1:**
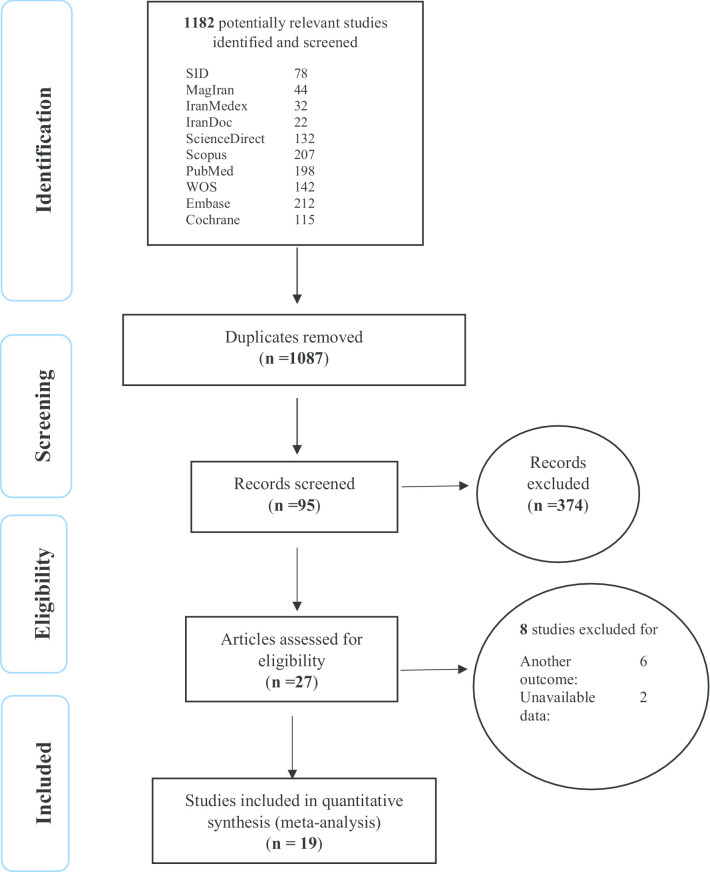
The PRISMA flow diagram of study selection

In this work, studies were reviewed in accordance with the Preferred Reporting Items for Systematic Reviews and Meta-Analyses (PRISMA) guidelines. Initially, 1182 articles were identified, and following different PRISMA phases, a number of articles were excluded. Finally, 19 studies were selected for meta-analysis (Fig. 1).

The total amalgamated sample size was 1948. The details of the systematically reviewed studies are provided in Table 1. All studies were clinical trials; Nine articles were written in English and 10 in Persian (Table 1).

Considering the available data, the standardized mean difference indices were used to determine the outcome of the effects of the studies. The standardized mean difference index was used in the meta-analysis of studies where results were reported using standard deviation of mean ± SD. The results of meta-analysis showed that there was heterogeneity between studies and pre- and post- intervention heterogeneity values were I^2^ = 99.9 and I^2^ = 99.9 respectively; thus, the random effects method was used to amalgamated the reported results of the selected studies and determine an overall outcome.

**Fig. 2:**
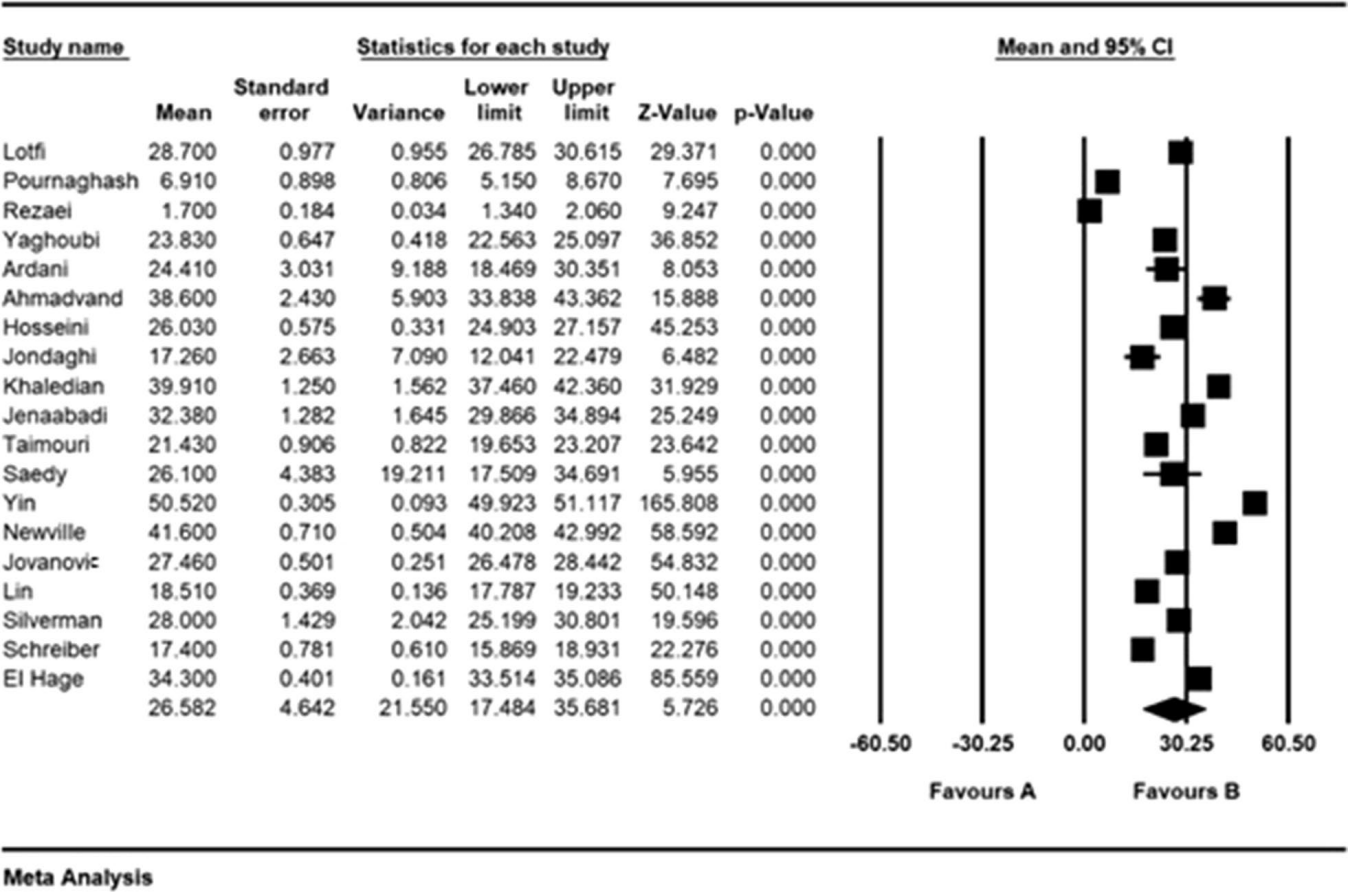
Accumulation diagram of studies included in the meta-analysis, using the standardized mean difference index before intervention

**Fig. 3:**
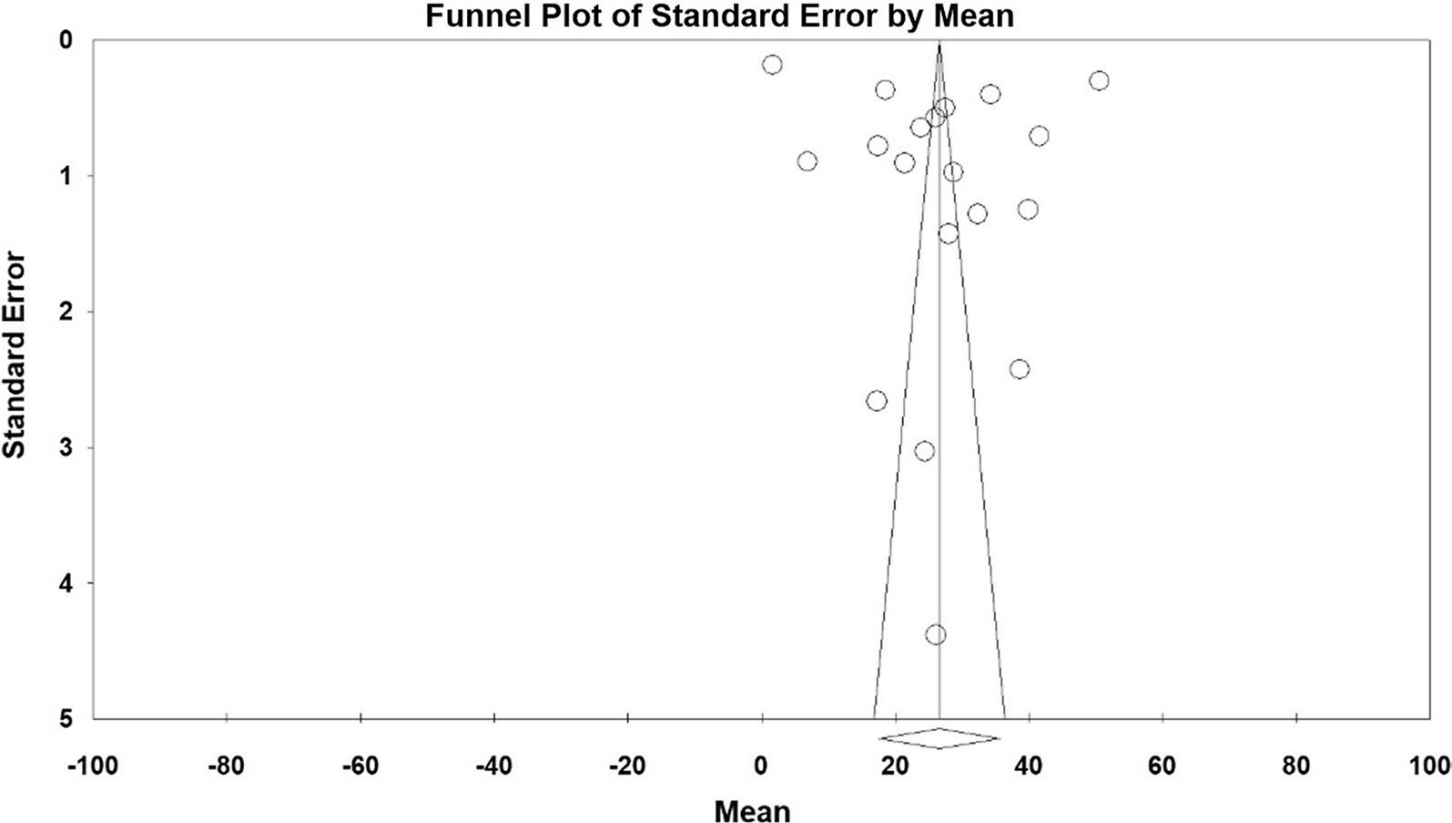
Funnel plot for the studies included in the meta-analysis before intervention

**Fig. 4:**
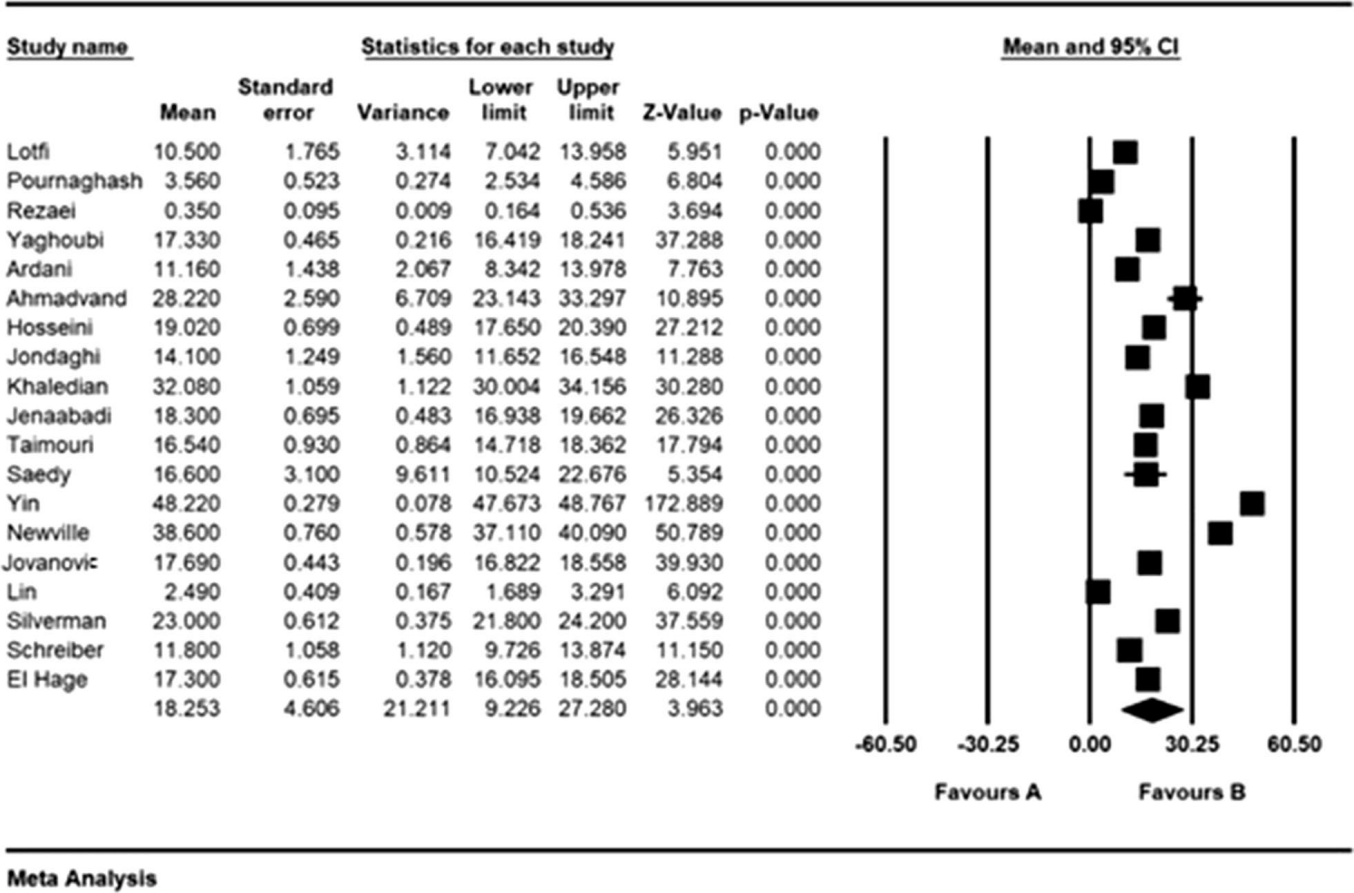
Accumulation diagram for the studies included in the meta-analysis using the standardized mean difference index after intervention

**Fig. 5:**
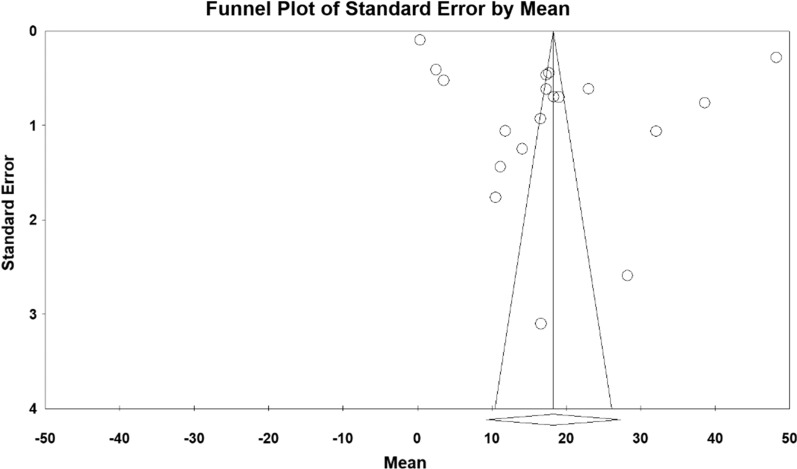
Funnel plot for the studies included in the meta-analysis after intervention

According to the results of the meta-analysis, the pre- and post- intervention standardized mean difference in the intervention group was estimated to be 26.5 ± 4.6 and 18.2 ± 4.6, respectively, indicating that methadone reduces depression. The accumulation diagrams (Figs. 2 and 3) show the standardized mean difference index and 95% confidence interval in each study as well as the final estimation of the index obtained from the combination of all results. The diagram demonstrates the weight of each study in the final combined value, and the size of each square is proportional to the weight of each study in the meta-analysis. The horizontal line of each square also denotes a 95% confidence interval. The Egger's test was used to investigate the publication bias in the articles, and the results showed no publication bias in the pre-and post-intervention studies (*P* = 0.184, *P* = 0.052, respectively) (Figs. 4 and 5).

### Sub-group analysis

According to many studies conducted in Iran, subgroup analysis was performed for studies in Iran and other countries. according to the results of the meta-analysis in Iran, the pre- and post- intervention standardized mean difference in the intervention group was estimated to be 23.9 ± 4.6 and 15.5 ± 3.2 and according to the results of the meta-analysis in other countries, the pre- and post- intervention standardized mean difference in the intervention group was estimated to be 27.8 ± 3.8 and 18.4 ± 4.8, respectively (Table 2).

**Table 2:** Subgroup analysis for studies in Iran and other countries

Countries	Number of articles	Sample size	Heterogeneity (I^2^)	Egger’s test	Mean ± SD of before	Mean ± SD of after
Iran	12	327	99.7	0.053	23.9 ± 4.6	15.5 ± 3.2
Other countries	7	1621	99.6	0.751	27.8 ± 3.8	18.4 ± 4.8

**Fig. 6:**
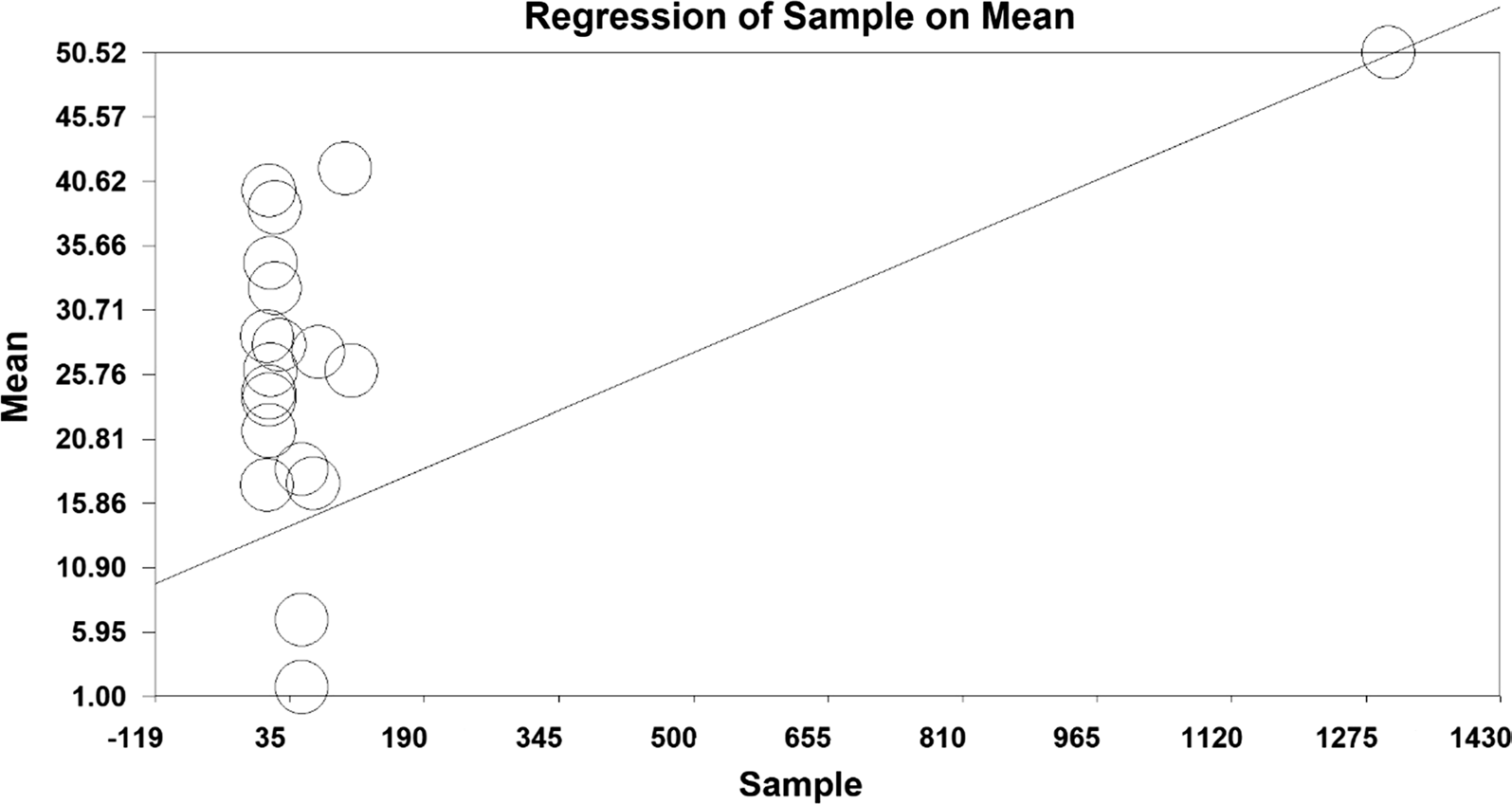
Investigating the heterogeneity of studies reviewed before intervention based on sample size

**Fig. 7:**
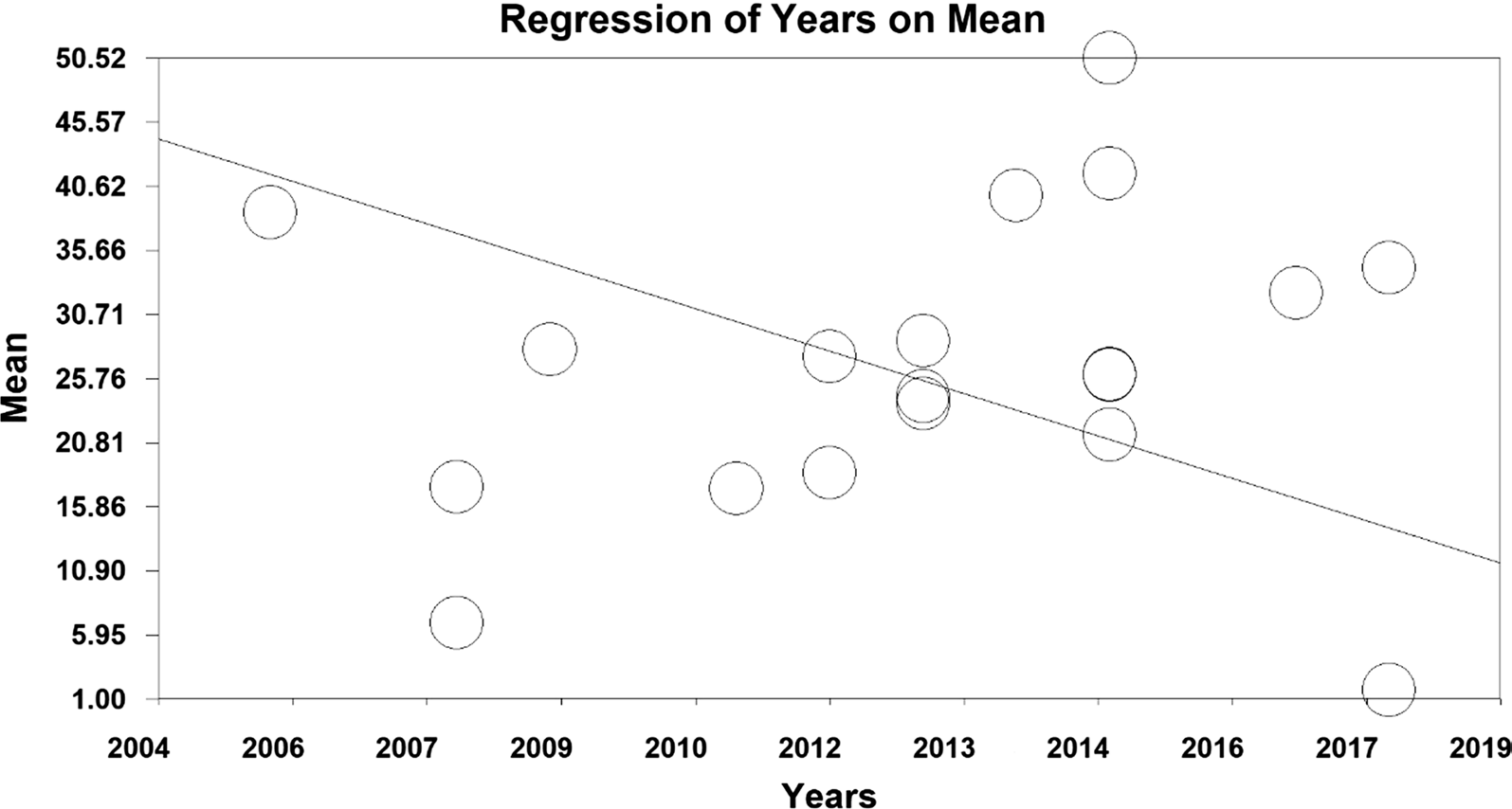
Investigating the heterogeneity of studies reviewed before intervention based on the year of study

**Fig. 8:**
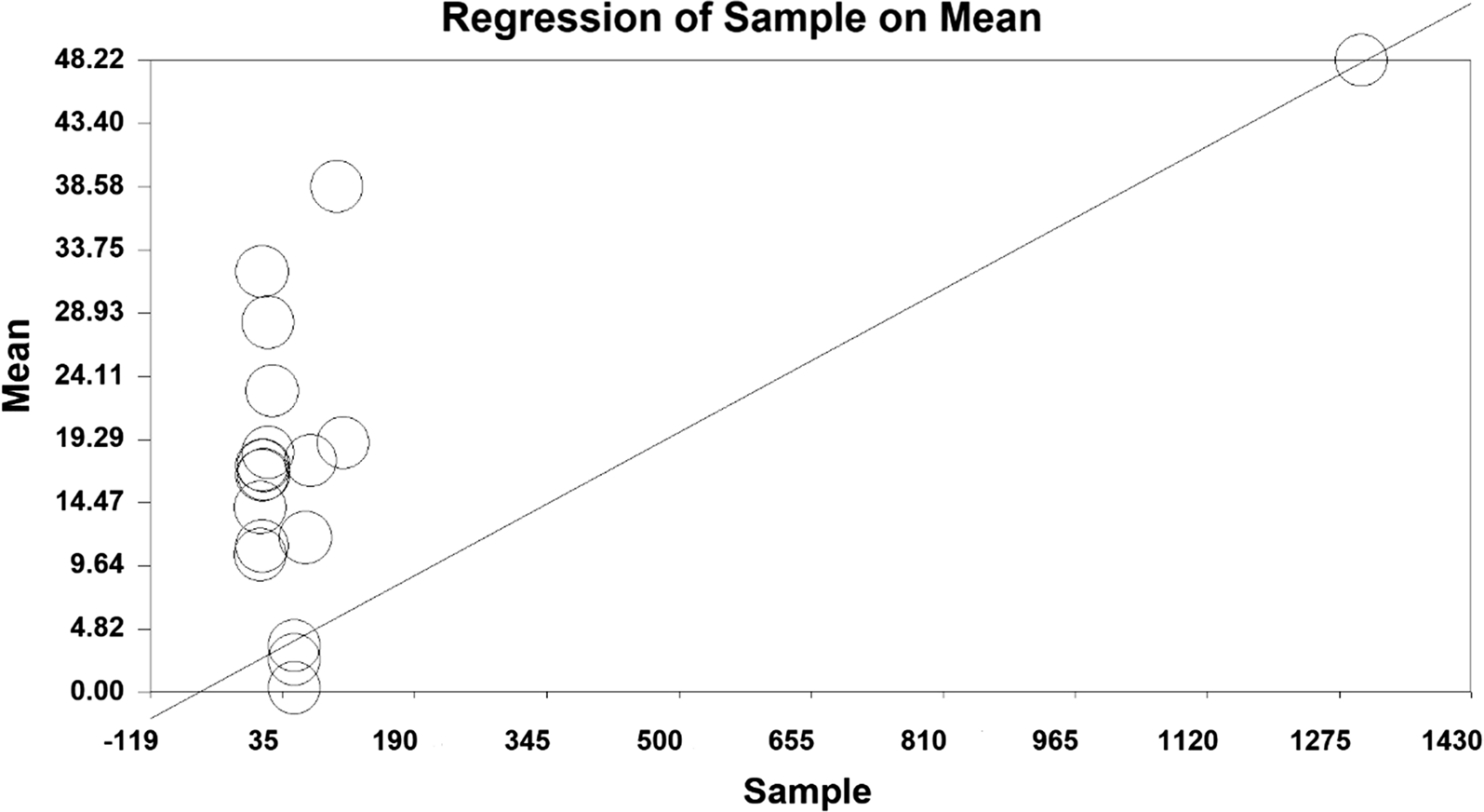
Investigating the heterogeneity of studies reviewed after intervention based on sample size

**Fig. 9:**
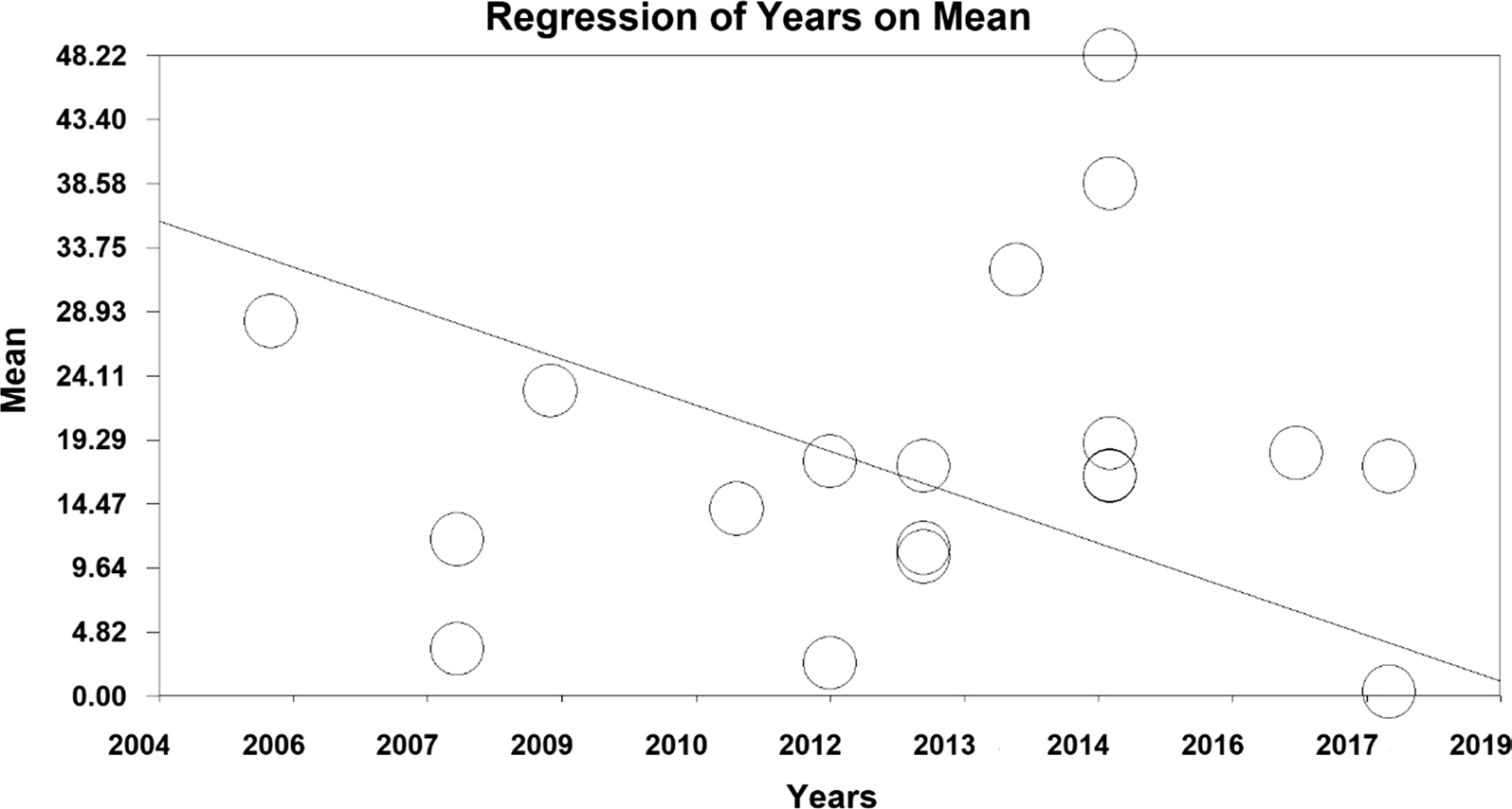
Investigating the heterogeneity of studies reviewed after intervention based on the year of study

In order to investigate the effect of heterogeneity factors, meta-regression test was used to determine which factor has the greatest impact on the values of meta-analysis. The two factors of ‘sample size’ and ‘year of publication’ were considered in the meta-regression. Results showed that with increasing sample size, mean depression scores increased, whilst mean depression decreased with increasing year of study before intervention (Figs. 6 and 7). Moreover, the mean depression scores increased with increasing sample size, and the mean depression score decreased with increasing years of study after the intervention (Figs. 8 and 9).

## Discussion

The lifetime prevalence of substance abuse is about 20% [34]. Nowadays, since drug abuse is one of the most important health problems in societies, health promotion practitioners are still in search of solutions to combat addiction and the associated problems. The aim of this study was thus to determine the effect of methadone on depression in addicts in Iran and around the world using a meta-analysis approach.

The results of this study show that the prevalence of depression among addicts is high, which indicates the impacts of addiction on all aspects of addicts' lives. Therefore, it is necessary to intervene in lifestyle changes, offer regular control of depression in people to prevent the disorder, and reduce its complications. In addition, since depression is primarily preventable and can be controlled and treated in the event of a complication, it is necessary to provide people with complete education about depression and how to prevent its complications, as well as early and timely diagnosis of the complications. Due to the high prevalence of depression among addicts, it is recommended that physicians pay more attention to the symptoms of this disorder among drug abuse patients; education should also be offered through various suitable media with the aim of raising awareness, and to reduce delays in the diagnosis.

The results also show a significant difference between the mean scores of depression severity in the intervention group before and after intervention. The results of the present study reported a mean depression severity of 26.4 ± 5.6 in the patients of the intervention group in the pretest phase, while it was significantly reduced to 18.2 ± 4.6 in the posttest phase.

The most comprehensive study in terms of sample size was the work of Yin et al. [27] in China, which reported the mean severity of depression in the pretest phase as 50.52 ± 10.99 and 48.22 ± 10.06 in the posttest. Furthermore, one of the most high-quality research works (according to the CONOSRT checklist) was conducted by El Hage et al. in Canada [33] which reported the mean severity of depression in the pre-test 34.3 ± 1.5 and the post-test 17.3 ± 2.3; the results are consistent with the overall results of our work.

Depression is characterized by symptoms such as helplessness and hinders the cessation of addiction and the use of available coping resources. However, the results of some studies suggest that treatment of addiction-related mood disorders may decrease the onset and recurrence [35].

Methadone is one of the medications used to treat addiction. Methadone maintenance treatment was proposed by Vincent Dole and Marie Nyswander in 1964, with the hypothesis that high methadone doses can eliminate the thirst for substance use and interrupt any euphoria of self-heroin resulting from cross tolerance. Thus, opiate users are free from mental occupation with substance-seeking and substance-seeking behaviors, and their body enzymes can therefore be redirected to more efficient pathways [36].

There have been very few studies comparing the efficacy of methadone maintenance therapy and other common treatments, including non-pharmacological treatments, on the psychiatric disorders of the population of drug abusers. Evidences from reviewed experimental studies suggest the efficacy of opioid dependence and agonist maintenance therapies, especially methadone maintenance therapy, as a common treatment in most countries [37].

One of the characteristics that makes methadone suitable for the addiction treatment is the efficacy of this drug in eliminating the withdrawal symptoms, that are occurring due to suspension of drugs intake for 24 h or longer. Methadone is a long-acting pure agonist that resides on μ receptors and removes withdrawal symptoms [38].

Broers et al. referred to the role of methadone as a protective factor in reducing injections and common syringe use [39]; similarly, Beoers et al. highlighted the role of methadone treatment in significantly reducing AIDS and hepatitis B and C among addicts [40]. Dolan et al. also reported that methadone therapy (over 60 mg/day) is effective in reducing injections in addicts [41].

In a study comparing two groups of methadone-treated addicts (n = 62), Lollis et al. concluded that methadone-treated groups had less risky sexual partners and were more likely to use safe sex, and there was a significant difference between the two groups in this regard [42]; their findings are consistent with the results of the research work conducted by Gossop et al. [43].

To investigate the effect of factors affecting heterogeneity, the meta-regression test was used for the two factors of ‘sample size’ and ‘year of the study’. The results show that before and after the intervention, with increasing sample size, the average depression scores increase. Moreover, before and after the intervention, with increasing year of study, the average scores of depression decrease, which may be due to the development of treatment and diagnostic methods for depression in recent years.

Methadone use clearly reduces risky behaviors such as injections, tattoos, self-harm, unsafe sexual behaviors, and imprisonment. Given the prevalence of addiction in communities and the detrimental consequences of such high-risk behaviors, particularly transmission of infections such as AIDS, hepatitis B and C, and the associated costs, the development of addiction treatment centers, encouragement of addicts to use therapies offered at these centers, and provision of related facilities are recommended.

### Limitations

One of the limitations in some of the articles was the inability to protect the anonymity of patients and therapists, which was not possible due to the nature of oral methadone treatment.

Another limitation of the study was that some samples were not randomly selected. Moreover, among the limitations, the non-uniform reporting of articles, inconsistent implementation methods, and the unavailability of the full text of the papers that has been presented at conferences can be mentioned.

## Conclusion

The results of the present study show that methadone significantly reduces depression among substance abusers. It is recommended to treat addicts using methadone under the supervision of a psychiatrist and along with counseling in addiction treatment centers.

## Data Availability

Datasets are available through the corresponding author upon reasonable request.

## Abbreviations

SID: Scientific Information Database
DSM-IV-TR: Diagnostic and Statistical Manual of Mental Disorders, Fourth Edition, Text Revision
MMT: Methadone Maintenance Treatment
PRISMA: Preferred Reporting Items for Systematic Reviews and Meta-Analysis

## References

[1] Farhadinasab A, Manikashani KH (2008). Substitution therapy with methadone and evaluation of depression in drug users in Hamadan. J Ilam Univ Med Sci.

[2] Asayesh H, Roohi G, Rahmani H, Nasiri H, Abbasi A (2008). The role of family in preventing from addiction in adolescents. J Gorgan Univ.

[3] Heidari J, Jafari H, Hosseini SH, Janati Y, Mohammad Pour RA, Mahmoudi G (2006). Study on the psychosocial conditions of addicts in Sari Township. J Mazandaran Univ Med Sci.

[4] Tawakoli-Ghuchani H, Shojaeizadeh D, Mazloum SR (2009). Comparative Study of factors associated with addiction withdrawal in clients referring to drug-stop clinics of northern Khorasan (Iran) in 2007. J Ilam Univ Med Sci.

[5] Ghaleiha A, Farhadi-Nasab A, Zarabian MK, Matinnia N (2008). Comparative survey of mental disorders and personality characteristics in persons with drug dependent and non drug dependent in Hamadan. J Hamadan Univ Med Sci.

[6] Kaplan H, Sadock B, editors. Synopsis of psychiatry, behavioral sciences, clinical psychiatry, 7 th ed. New York: WB Saunders; 2006.

[7] Sadock, BJ, Sadock VA,Ruiz P. Comprehensive textbook of psychiatry. Lippincott Williams & Wilkins Philadelphia; 2000.

[8] Dolan SL, Martin RA, Rohsenow DJ (2008). Self-efficacy for cocaine abstinence: pretreatment correlates and relationship to outcomes. Addict Behav.

[9] Afariny Y, Hosseini S (2018). The effect of psychodrama on the treatment of depression in improved drug-addicted patients. Horizon Med Sci.

[10] McGovern MP, Fox TS, Xie H, Drake RE (2004). A survey of clinical practices and readiness to adopt evidence-based practices: dissemination research in an addiction treatment system. J Subst Abuse Treat.

[11] Khodabandeh S, Loripoor M, Hossinrezaei H (2008). The comparison religious and familial characteristics between abuser drug quiting addiction in Mahyar rehabilitation center of Isfahan and control group. J Rafsanjan Uni Med Sci.

[12] Karami S, Jaafari M (2008). The effect of cognitive behavioral education on reduce the amount of depression addicts (TC) located in the center of the therapeutic community. J Drug Abuse.

[13] Dobson KS, Mohammad K (2006). Psychometric characteristics of the beck depression inventory in patients with major depressive disorder in partial remission. Soc Welfare Rehab Sci.

[14] Alpoim PN, de Barros Pinheiro M, Junqueira DR, Freitas LG,das Gracas Carvalho M, Fernández AP, et al. Preeclampsia and ABO blood groups: a systematic review and metaanalysis. Mol Biol Rep. 2012; 40: 2253–61. 10.1007/s11033-012-2288-2 23184045

[15] Lotfi F, Mojtabaei M, Ali Mahdi M. Comparison of the effects of cognitive behavioral therapy, methadone therapy and combination therapy on depression in addicts treated, Knowledge and Research in Applied Psychology. Year 2013; 4, Number 4, pp 19–26. (**In Persian**)

[16] Pournaghash S (2008). Comparison of the efficacy of methadone maintenance therapy and methadone detoxification treatment on symptoms of anxiety and depression in drug-dependent individuals, Faculty of Educational Sciences and Psychology. Alzahra Univ. Period.

[17] Rezaei Z, Rasoli-Azad M, Mehrzad F, Farhad M, Azad-Miveh Z. The effect of dialectical behavioral therapy on craving and depression in methadone patients. J Kashan University of Medical Sciences, 2019; Vol. 22, No 6, 602–9. (**In Persian**)

[18] Yaghoubi E, Basak nejat S, Mehrabi zadeh M, Zamiri Nejad S. (2013). The effectiveness of meta-cognitive group therapy on depression symptoms in methadone maintenance addicts. J North Khorasan Univ Med Sci 5(1): 167–174. (**In Persian**)

[19] Ardani AR, Erfanian Taghvaei Yazdi-Nejad Z, Shayesteh Zarrin M. The effect of cognitive-behavioral group therapy using trans-theoretical model on the level of depressive symptoms and methadone dose consumed by patients undergoing methadone maintenance treatment, 2013; Vol. 15, No. 2(58), 91–9. (**In Persian**)

[20] Ahmadvand A, Ghorashi F, Sepehr Manesh Z, Mousavi S Gh. The effect of methadone on depression in prisoner injecting addicts. J Behav Sci Res. 2013; the period 4, number 1, pp. 77–82. (**In Persian**)

[21] Hosseini M, Hashemi R. (2015). Addicts' quality of life and psychological disorders (depression, anxiety, and stress) in two treatment methods: narcotics anonymous vs. methadone maintenance treatment. quality of life. Q J Res Addict **9**(35). 119–136 (**In Persian**)

[22] Jondaghi F, Neshat Doust H, Kalantari M, Jabalameli Sh. The effectiveness of cognitive-behavioral stress management group training on anxiety. Depression in people with methadone maintenance drug abuse. J Clin Psychol. Year 2011; 4, Number 4, pp 41–50. (**In Persian**)

[23] Khaledian M, Kmrzrin H, Jalalian A (2014). The effectiveness of cognitive-behavioral group therapy on depression in addicts. Q J Res Addict.

[24] Jenaabadi H, Jahangir AH (2017). Comparing the effectiveness of mindfulness-based group therapy and methadone maintenance therapy on psychological symptoms (obsession, interpersonal sensitivity, depression, anxiety, and aggression) among opioid-dependent patients. Shiraz E-Med J.

[25] Taimouri S, Ramazani F, Mahbob N (2015). Effectiveness of mindfulness-based therapeutic effectiveness on reducing rumination and depression in methadone depressed women. Q J Res Addict.

[26] Saedy M, Kooshki S, Firouzabadi MJ, Emamipour S, Ardani AR. 2015. Effectiveness of acceptance-commitment therapy on anxiety and depression among patients on methadone treatment: a pilot study. Iran J Psychiatry Behav Sci 9(1). 10.17795/ijpbs222 PMC4525449 26251660

[27] Yin W, Pang L, Cao X, McGoogan JM, Liu M, Zhang C, Li Z, Li J, Rou K (2015). Factors associated with depression and anxiety among patients attending community-based methadone maintenance treatment in China. Addiction.

[28] Newville H, Berg KM, Gonzalez JS (2015). The interaction of active substance use, depression, and antiretroviral adherence in methadone maintenance. Int J Behav Med.

[29] Jovanović T, Lazarević D, Nikolić G (2012). Differences in depression severity and frequency of relapses in opiate addicts treated with methadone or opiate blocker after detoxification. Vojnosanit Pregl.

[30] Lin W-C, Chou K-H, Chen H-L, Huang C-C, Lu C-H, Li S-H, Wang Y-L, Cheng Y-F, Lin C-P, Chen C-C (2012). Structural deficits in the emotion circuit and cerebellum are associated with depression, anxiety and cognitive dysfunction in methadone maintenance patients: a voxel-based morphometric study. Psychiatry Res: Neuroimaging.

[31] Silverman DA, Nettleton RT, Spencer KB, Wallisch M, Olsen GD (2009). S-methadone augments R-methadone induced respiratory depression in the neonatal guinea pig. Respir Physiol Neurobiol.

[32] Schreiber S, Peles E, Adelson M (2008). Association between improvement in depression, reduced benzodiazepine (BDZ) abuse, and increased psychotropic medication use in methadone maintenance treatment (MMT) patients. Drug Alcohol Depend.

[33] El Hage C, Ghabrash MF, Dubreucq S, Brissette S, Lespérance F, Lespérance P, Ouellet-Plamondon C, Bruneau J, Jutras-Aswad D (2018). A pilot, open-label, 8-week study evaluating desvenlafaxine for treatment of major depression in methadone-maintained individuals with opioid use disorder. Int Clin Psychopharmacol.

[34] Sepehrmanesh Z, Ahmadvand A, Ghoreyshi F, Mousavi G (2008). Personality traits of IV drug abusers of Kashan prison. Feyz J.

[35] Fitzsimons HE, Tuten M, Vaidya V, Jones HE (2007). Mood disorders affect drug treatment success of drug-dependent pregnant women. J Subst Abuse Treat.

[36] Akbari B (2009). Relationship between commitment to prayer and anxiety, according to sociodemographic variables among students of Islamic Azad University Anzali. J Psychol Religion.

[37] Drummond, D. C. and K. Perryman (2007). Psychosocial interventions in pharmacotherapy of opioid dependence: a literature review. London, St George's University of London, Division of Mental Health, Section of Addictive Behaviour.

[38] Carol MC, Onken LS. Behavioral therapyes for substance abuse. 2008: (4)5: 153–5.

[39] Broers B, Junet C, Bourquin M, Déglon J-J, Perrin L, Hirschel B (1998). Prevalence and incidence rate of HIV, hepatitis B and C among drug users on methadone maintenance treatment in Geneva between 1988 and 1995. Aids.

[40] Dolan KA, Wodak AD, Hall WD (1998). Methadone maintenance treatment reduces heroin injection in New South Wales prisons. Drug Alcohol Rev..

[41] Lollis CM, Strothers HS, Chitwood DD, McGhee M (2000). Sex, drugs, and HIV: does methadone maintenance reduce drug use and risky sexual behavior?. J Behav Med.

[42] Gossop M, Marsden J, Stewart D, Treacy S (2002). Reduced injection risk and sexual risk behaviours after drug misuse treatment: results from the National Treatment Outcome Research Study. AIDS care.

[43] Kaplan HI, Saddock BJ, Grebb JA. Comprehensive textbook of psychiatry, 7th ed. William and Wilkins; 1999, 1055–7.

